# Short-term effects of Chlorhexidine mouthwash and Listerine on oral microbiome in hospitalized patients

**DOI:** 10.3389/fcimb.2023.1056534

**Published:** 2023-02-02

**Authors:** Tsunglin Liu, Yen-Chin Chen, Shuen-Lin Jeng, Jui-Jen Chang, Jiu-Yao Wang, Cheng-Han Lin, Pei-Fang Tsai, Nai-Ying Ko, Wen-Chien Ko, Jiun-Ling Wang

**Affiliations:** ^1^ Department of Biotechnology and Bioindustry Sciences, National Cheng Kung University, Tainan, Taiwan; ^2^ Department of Nursing, National Cheng Kung University Hospital, Tainan, Taiwan; ^3^ Department of Nursing, National Cheng Kung University, Tainan, Taiwan; ^4^ Department of Statistics, Institute of Data Science, Center for Innovative FinTech Business Models, National Cheng Kung University, Tainan, Taiwan; ^5^ Graduate Institute of Integrated Medicine, Department of Medical Research, China Medical University Hospital, Taichung, Taiwan; ^6^ Center of Allergy, Immunology and Microbiome (AIM), Department of Allergy and Immunology, China Medical University Children’s Hospital, Taichung, Taiwan; ^7^ Department of Pathology, National Cheng Kung University Hospital, College of Medicine, National Cheng Kung University, Tainan, Taiwan; ^8^ Department of Internal Medicine, National Cheng Kung University Hospital, Tainan, Taiwan; ^9^ Department of Medicine, College of Medicine, National Cheng Kung University, Tainan, Taiwan

**Keywords:** mouthwash, Chlorhexidine, essential oil, oral microbiome, Listerine antiseptic mouth rinse

## Abstract

**Introduction:**

Chlorhexidine (CHX) and essential oil containing mouthwashes like Listerine^®^ can improve oral hygiene *via* suppressing oral microbes. In hospitalized patients, CHX mouthwash reduces the incidence of ventilator-associated pneumonia. However, CHX use was also associated with increased mortality, which might be related to nitrate-reducing bacteria. Currently, no study determines oral bacteria targeted by essential oils mouthwash in hospitalized patients using a metagenomic approach.

**Methods:**

We recruited 87 hospitalized patients from a previous randomized control study, and assigned them to three mouthwash groups: CHX, Listerine, and normal saline (control). Before and after gargling the mouthwash twice a day for 5-7 days, oral bacteria were examined using a 16S rDNA approach.

**Results:**

Alpha diversities at the genus level decreased significantly only for the CHX and Listerine groups. Only for the two groups, oral microbiota before and after gargling were significantly different, but not clearly distinct. Paired analysis eliminated the substantial individual differences and revealed eight bacterial genera (including *Prevotella*, *Fusobacterium*, and *Selenomonas*) with a decreased relative abundance, while *Rothia* increased after gargling the CHX mouthwash. After gargling Listerine, seven genera (including *Parvimonas*, *Eubacterium*, and *Selenomonas*) showed a decreased relative abundance, and the magnitudes were smaller compared to the CHX group. Fewer bacteria targeted by Listerine were reported to be nitrate-reducing compared to the CHX mouthwash.

**Discussion:**

In conclusion, short-term gargling of the CHX mouthwash and Listerine altered oral microbiota in our hospitalized patients. The bacterial genera targeted by the CHX mouthwash and Listerine were largely different and the magnitudes of changes were smaller using Listerine. Functional alterations of gargling CHX and Listerine were also different. These findings can be considered for managing oral hygiene of hospitalized patients.

## 1 Introduction

Therapeutic mouthwash can improve oral hygiene, e.g., by reducing dental plaque and gingivitis (Araujo et al., 2015; Van der Weijden et al., 2015; Brookes et al., 2020). Dental plaque is a biofilm of oral microbes, mostly bacteria that grow on teeth. Build-up of plaque can lead to dental decay and gingival inflammation (Loesche, 1996).

The anti-plaque activity of mouthwash is partly attributed to its antimicrobial capability, and the antiseptic ingredients include chlorhexidine (CHX) and essential oils. CHX is a potent antimicrobial agent and is widely used as disinfectant in various medical fields, such as dermatology and surgery (Lim and Kam, 2008). CHX kills bacteria *via* binding and perforating cell membranes (Cheung et al., 2012). Antimicrobial activity of essential oils mouthwash has also been documented (Chouhan et al., 2017). The popular mouthwash brand Listerine^®^ (LIS), which contains four plant-derived essential oils (eucalyptol, menthol, methyl salicylate, thymol), has also been shown to reduce plaque and gingivitis (Alshehri, 2018).

Besides its dental application, CHX mouthwash was suggested to reduce the risk of developing ventilator-associated pneumonia in critically ill patients (Hua et al., 2016). The benefit, however, might apply only to cardiac surgery patients (Klompas et al., 2014), and was further argued in a study of hospitalized patients that CHX mouthwash was associated with increased mortality (Deschepper et al., 2018). The association was hypothesized to stem from an obliteration of oral nitrate-reducing bacteria (such as *Veillonella*, *Prevotella*, *Neisseria*, and *Haemophilus*), which might cause nitric oxide deficiency (Hyde et al., 2014; Blot, 2021) and the subsequent ischemic heart events or sepsis. Because of the uncertainty in risk-benefit balance, current guidelines provide no formal recommendation for using CHX for oral care of hospitalized patients (Tran and Butcher, 2019). The pros and cons of using other mouthwash for the patients’ oral care is not yet reported to our knowledge. These show the importance of identifying bacterial species targeted by different mouthwashes.

Bacterial species altered by CHX mouthwash have been studied *via* examining *in vitro* communities of oral microbes (McBain et al., 2003; Eick et al., 2011). For example, exposing an *in vitro* community of ten oral bacterial species to CHX resulted in marked reductions in *Prevotella* sp. and *Selenomonas infelix*, while other species were reduced to a lesser degree (McBain et al., 2003). The effect of LIS on bacterial species has also been examined using a culturing approach for quantifying bacterial levels (Fine et al., 2007). In the study, *Veillonella* sp., *Capnocytophaga* sp., and *Fusobacterium nucleatum* in subgingival plaque were reduced significantly after gargling LIS for two weeks. In another *in vitro* study of 40 oral bacterial species, CHX mouthwash was more effective than LIS mouthwash in reducing all the species (Haffajee et al., 2008).

Although these *in vitro* studies were informative, the results may not reflect the *in vivo* dynamics of all oral microbes. A metagenomic approach, e.g., *via* examining 16S rRNA genes, is powerful for exploring environmental microbes. The approach, however, has only been applied in a few studies investigating oral microbes in the context of mouthwash. Tribble et al. studied tongue microbiome and found that *Leptotrichia* sp. and *Veillonella rogosae* were reduced on tongue after gargling CHX mouthwash for a week (Tribble et al., 2019). Bescos et al. examined the effects of CHX mouthwash on salivary microbiome of healthy individuals (Bescos et al., 2020). After gargling the mouthwash for a week, several bacterial genera, including *Prevotella*, *Antinomyces*, and *Fusobacterium*, were reduced, while the genera *Neisseria*, *Streptococcus*, etc., were increased. To our best knowledge, the effects of LIS on salivary microbiome using a 16S approach have not been reported, although the long term (12 weeks) effects of LIS on oropharyngeal microbiome was recently described (Plummer et al., 2022).

Here, we study the short-term effects of CHX mouthwash and LIS on oral microbiome of hospitalized patients using a 16S approach. We ask if the CHX and LIS mouthwash lead to different dysbiosis of oral bacteria. The findings can be considered for managing oral hygiene of patients at the internal medicine ward.

## 2 Materials and methods

### 2.1 Study design and oral sample collection

Hospitalized patients were recruited for a clinical trial about the effects of CHX oral rinse on preventing hospital-acquired pneumonia in a general ward (Clinical Trials IRCT ID: NCT04403971)(Chen et al., 2022). In the trial, only patients who were aged ≧50 years, could rinse orally, and communicated well were included. The exclusion criteria were: an inpatient hospital stay of less than three consecutive calendar days and admission for routine or quick examination of acute psychiatric syndromes, chlorhexidine allergy, and cardiac catheterization. The main admission diagnoses were infectious diseases (44%) and gastrointestinal diseases (32%).

Patients were randomly assigned to three mouthwash groups: CHX (0.12%; aseptic innovative medicine co., ltd, Taipei, Taiwan), LIS (total care anticavity fluoride mouthwash), and normal saline. One research investigator who qualified for oral care training was blind to the group allocation evaluated the oral health status by using the oral health assessment tool (Chalmers et al., 2005).The tool included the eight subscales of lips, tongues, gums and tissues, saliva, natural teeth, dentures, oral cleanliness, and dental pain. The intraclass correlation coefficients for oral health assessment tool were set as 0.78 for intra-carer and 0.74 for inter-carer reliability (Chalmers et al., 2005).

According to a previous study (Chen et al., 2022), a sample size about 30 individuals is sufficient for studying oral microbiota. We recorded the incidence of hospital-acquired pneumonia and changes in clinical pulmonary infection score among the three groups (Chen et al., 2022). The patients who provided oral microbial samples on Day 0 and Day 5-7 were included in this study. The mouthwash was placed in a cloudy container and each patient gargled the mouthwash twice a day (30-60 seconds with 10** **ml) for 5-7 days under the supervision of a nurse. The first oral microbial samples were collected from each patient before intervention (Day 0) and the second sample was collected on the day after the last day of the intervention (5-7 days after the first use of mouthwash). We collected the samples before breakfast and brushing teeth in the early morning. To collect a sample, the patient gargled 20 mL of 0.9% saline and then spat the fluid into a sterile container. We did not intervene in the toothpaste use and the method of brushing teeth.

### 2.2 DNA extraction and 16S rDNA sequencing

Collected liquid was processed by centrifugation at 1000 × g for 10 minutes to separate the cellular pellet from cell-free suspension (Lim et al., 2017). From the oral samples pellets, total DNA were extracted and preserved using the DNA/RNA Shield Saliva Collection Kit (Zymo Research), and the extracts was stored at -70∘C. Total genomic DNA from samples was extracted using the column-based method (QIAamp PowerFecal DNA Kit, Qiagen). For the 16S rRNA gene sequencing, V3-V4 region was amplified by specific primer set (319F: 5’-CCTACGGGNGGCWGCAG-3’, 806R: 5’-GACTACHVGGGTAT CTAATCC -3’) according to the 16S Metagenomic Sequencing Library Preparation procedure (Illumina). In brief, 12.5 ng of gDNA was used for the PCR reaction carried out with KAPA HiFi HotStart ReadyMix (Roche) under the PCR condition: 95°C for 3 minutes; 25 cycles of: 95°C for 30 seconds, 55°C for 30 seconds, 72°C for 30 seconds; 72°C for 5 minutes and hold at 4°C. The PCR products were monitored on 1.5% agarose gel. Samples with bright main strip around 500bp were chosen and purified by using the AMPure XP beads for the following library preparation. The Sequencing library was prepared according to the 16S Metagenomic Sequencing Library Preparation procedure (Illumina). A secondary PCR was performed by using the 16S rRNA V3-V4 region PCR amplicon and Nextera XT Index Kit with dual indices and Illumina sequencing adapters (Illumina). The indexed PCR product quality was assessed on the Qubit 4.0 Fluorometer (Thermo Scientific) and Qsep100TM system. Equal amount of the indexed PCR product was mixed to generate the sequencing library. At last, the library was sequenced on an Illumina MiSeq platform and paired 300‐bp reads were generated.

### 2.3 Ethics approval

The study was approved by the institutional review board of the National Cheng Kung University Hospital (no: A-ER-108-397). All patients provided informed consent.

### 2.4 Data preprocessing and clustering

Raw paired-end reads were merged using FLASH (v1.2.11; option: -M 300) (Magoc and Salzberg, 2011), and short (<400 bp) merged reads were discarded. The preprocessed reads of all samples were clustered into zero-radius operation taxonomy units (ZOTUs) using UNOISE3 with default options (in USEARCH v11.0.667) (Edgar, 2010; Edgar, 2016). In the UNOISE3 pipeline, reads were quality filtered and dereplicated. Dereplicated reads were clustered into ZTOU sequences with sequencing errors corrected and chimera removed. Merged reads were then aligned to the ZTOU sequences to obtain a ZOTU table showing ZOTU abundances of all samples.

### 2.5 Taxonomy annotation

ZOTU sequences were annotated by RDP classifier (v2.13) (Wang et al., 2007), which was retrained to include species information. Specifically, the reference data of trainset 18 from RDP classifier were preprocessed to remove redundant sequences and unite the annotation format (e.g., setting species name of uncultured or unspecified bacteria as “genus_name sp.”) for a retrain. An annotation with a confidence score <0.8 was considered unclassified. Based on the annotations by RDP classifier, eukaryotic ZOTUs were filtered. ZOTU table was also converted into taxon abundance and percentage table at different taxonomy levels. Percentages of the top 30 abundant taxa were visualized in a heatmap using “clustermap()” in the python package seaborn (v0.9), in which the hierarchical clustering was done with the average linkage method and braycurtis dissimilarity as the metric.

### 2.6 Diversity and statistical analysis

For a fair comparison, ZOTU abundances of all samples were rarefied to the lowest total read count for diversity analyses. Alpha diversity indices (e.g., Chao1, Shannon, and Simpson) were calculated based on taxon percentages at different levels using “alpha_diversity.py” of QIIME (v1.9) (Caporaso et al., 2010). The alpha diversities before and after gargling were compared using paired Wilcoxon rank test in R. For beta diversity analysis by QIIME (v2-2022.2) (Caporaso et al., 2010), the ZOTU sequences were aligned using the “phylogeny” plugin and “align-to-tree-mafft-fasttree” method, which produced a rooted tree. With the tree, ZOTU abundance table, and clinical descriptions of samples, beta diversity was calculated using the “diversity” plugin and “core-metrics-phylogenetic” method. The command generated UniFrac distances (Lozupone and Knight, 2005) for all pairs of samples, and the resulting principal coordinate analysis was visualized using R (v4.0.2).

Differences in microbial communities between groups of samples were tested using permutational multivariate ANOVA (PERMANOVA) in the vegan package (v2.5) of R based on the UniFrac distances (option: permutations=9999). Differentially abundant microbes after gargling were identified using MaAsLin2 (Mallick et al., 2021) in a paired manner. Before the analysis, taxa present in less than 10% of the samples were filtered. For paired analysis, taxa fractions after gargling were subtracted from those before gargling and the fractions before gargling were set zero. Each difference (d) in taxon frequency was further rescaled into range [0,1] (i.e., *via* (d+1)/2). For consistency, the taxa frequencies before gargling were shifted to 1/2. The processed frequencies were input into MaAsLin2 for analysis with the options: –normalization=NONE –transform=AST –analysis_method=LM –fixed_effects=“gargling” –random_effect=NONE –reference=“gargling,before” –correction=BH. An association with a q-value<0.1 was considered significant. Association between microbes and clinical features was also analyzed using MaAsLin2, and only samples before gargling were examined. The options remained similar except that all clinical features (i.e., sex, age, smoking, alcohol, BMI, diabetes, cancer, cirrhosis, ESRD) were set as fixed effects and the values of age and BMI were standardized.

### 2.7 Functional prediction and comparison

PICRUSt2 (v2.4.1) (Douglas et al., 2020) was used to predict microbiota function for each sample. First, ZOTU sequences were placed in the phylogenetic tree using EPA-NG (Barbera et al., 2019). For each ZOTU, gene family abundances and copy number of 16S genes were then predicted by the castor R package (Louca and Doebeli, 2018) using default options. With the predicted abundances and ZOTU abundances, functional gene abundances in the community were then estimated. Abundances of KEGG pathways (Kanehisa and Goto, 2000) identified as present by MinPath (Ye and Doak, 2009) were then inferred for the subsequent statistical analysis. We compared pathway abundance before and after gargling in a paired manner. Specifically, samples before and after gargling of the same individual were compared using STAMP (v2.1.3) (Parks et al., 2014). Differential abundance was tested using “G-test (w/Yate’s) + Fisher’s” and the p-values were corrected by Benjamin-Hochberg FDR. Abundance of a pathway was considered different if the q-value was <0.1, and the effect size was recorded for subsequent analysis.

Differentially abundant pathways were further labeled by sign of the effect size, where minus and plus signs indicated lower and higher relative abundance after gargling, respectively. For each mouthwash group, we first picked the pathways that showed a preferred direction of change in the abundance among all the individuals. Specifically, each pathway was subjected to two-tailed binomial test for a biased distribution of individuals showing a lower and higher abundance under a null hypothesis of equal probabilities in both directions. The binomial test p-values were corrected by FDR and pathways with a corrected q-value <0.2 were considered differentially abundant with consistency.

## 3 Results

### 3.1 Sample collection and microbiome sequencing

Eighty-seven hospitalized patients were recruited and their clinical features were documented (**Table S1**). Of the patients, 28, 29, and 30 were randomly assigned into three mouthwash groups: CHX (abbreviated as C), LIS (L), and normal saline (N; control group), respectively. In the previous trial data (Chen et al., 2022), the CHX group had a significant improvement in oral health assessment tool scores than the LIS and normal saline group after mouthwash (−1.20 in CHX group, −0.75 in Group LIS group, and 0.25 in control group). The incidence of hospital-acquired pneumonia and changes in clinical pulmonary infection score did not differ among the three groups (Chen et al., 2022).

From each patient, two oral samples were collected before and after gargling the mouthwash respectively, and the samples were subjected to 16S rDNA sequencing. MiSeq sequencing of the 174 samples generated 14.2 million raw paired-end reads; 95.6% of which could be merged into single reads (**Table S2**). Among the merged reads, the 13.5M reads longer than 400 bp were clustered into 49,190 ZOTUs. Of those, 189 belonged to *Zea mays* and were discarded. The 174 samples had 37k~130k reads for community analysis.

### 3.2 Overall oral microbiota

**Figure 1:**
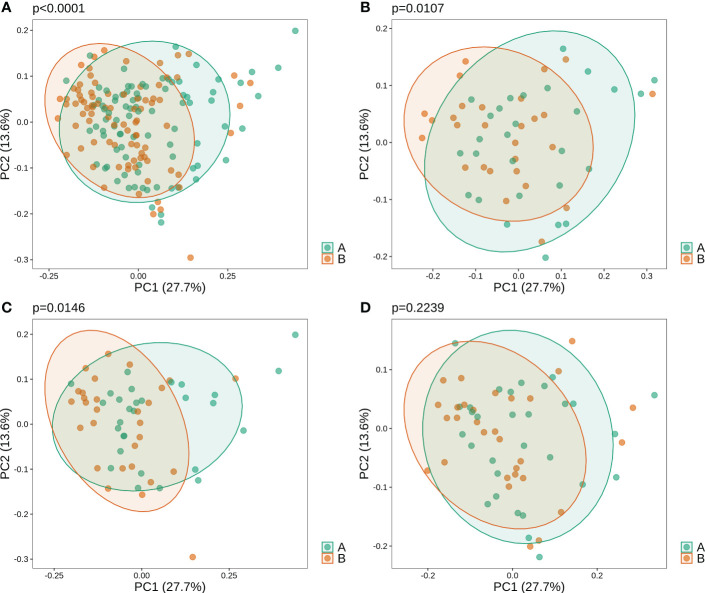
Weighted principal component analysis (PCoA) of oral microbiota for **(A)** all samples, and samples in three mouthwash groups: **(B–D)** C, L, and N. For each set of samples, the PERMANOVA p-value shows significance of difference in microbiota of samples before **(B)** and after **(A)** gargling.

Beta diversity analysis revealed that oral microbiota before and after gargling were significantly different (**Figure 1A**; PERMANOVA p-value<1*10^-4^). The difference in microbiota after gargling was also significant for mouthwash groups C and L respectively, but not for group N (**Figures 1B–D**). This shows that CHX and LIS mouthwashes altered oral microbiota. The alternation, however, was not strong enough to separate the microbiota before and after gargling, which still overlapped to a large extent.

**Figure 2:**
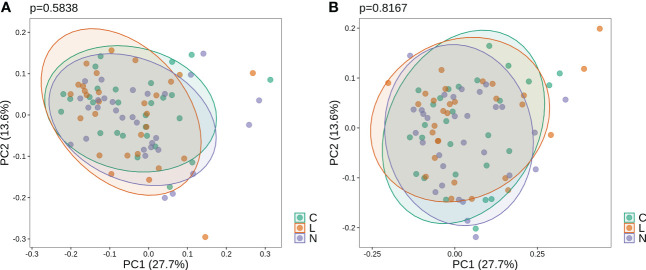
Weighted PCoA analysis of oral microbiota for samples **(A)** before and **(B)** after gargling. For each set of samples, the difference between the three mouthwash groups is quantified by the p-value.

Before gargling, oral microbiota between three mouthwash groups were not different (**Figure 2A**), which echoed our random assignment of patients into groups. After gargling, oral microbiota of the three groups were still not different (**Figure 2B**). This indicates that the microbial alternations after gargling were not strong enough to stand out from the overall difference among the three groups.

At the phylum level, the top three abundant taxa across all oral samples were Firmicutes, Bacteroidetes, and Proteobacteria (**Figure S1**). In terms of phyla composition, again we observed no clear distinction between the three groups, both before and after gargling. In the following, oral microbiota in samples of each mouthwash group were examined.

### 3.3 Oral microbiota of the CHX group

**Figure 3:**
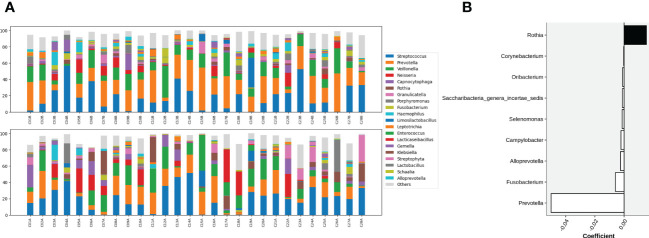
**(A)** Compositions of top 20 abundant genera in oral samples of group C before (top) and after (bottom) gargling. Rest genera are combined in the “Others” category. The space between 100% and height of each stacked bar shows the fraction of unclassified bacteria. **(B)** Differentially abundant genera; the +/- sign of a coefficient indicates an increase/decrease in the fraction after gargling and the value indicates the magnitude of change.

At the genus level, microbial composition across individuals spanned a large spectrum both before and after gargling (**Figure 3A**), which indicates substantial individual differences. A relatively large change in microbial composition after gargling was observed for several individuals.

**Figure 4:**
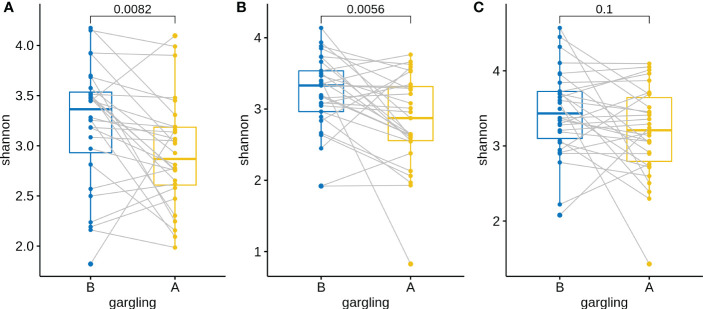
Shannon indices of oral microbiota in paired samples before **(B)** and after **(A)** gargling of mouthwash groups **(A)** C, **(B)** L, and **(C)** N. For each group, paired Wilcoxon rank test p-value is shown.

For identifying differentially abundant microbes after gargling, paired analysis was conducted to eliminate individual differences. The paired comparison revealed four differentially abundant phyla (**Table S3**) and nine differentially abundant genera (**Figure 3B**). Relative abundance of the four phyla (Fusobacteria, Campilobacterota, Candidatus Saccharibacteria, and Bacteroidetes) decreased after gargling. Of the nine genera, eight showed a decreased fraction and only *Rothia* increased after gargling. Among the decreased genera, the magnitude of decrement (i.e., the coefficient value in **Figure 3B**) was the largest for *Prevotella*. The species composition revealed that *Rothia mulcilaginosa* was the major species within the *Rothia* genus (**Figure S2**). In contrast, the *Prevotella* genus was composed of several species, e.g., *Prevotella melaninogenica, veroralis, oris, salivae*, etc. Note that only four of the nine differentially abundant genera were among the top 20 abundant genera, thus the rest five were absent from the figures. In the paired comparison, the diversity of genus composition decreased after gargling for 21 of the 28 patients in group C (**Figure 4A**) and the overall decrease was significant.

### 3.4 Oral microbiota of the LIS group

**Figure 5:**
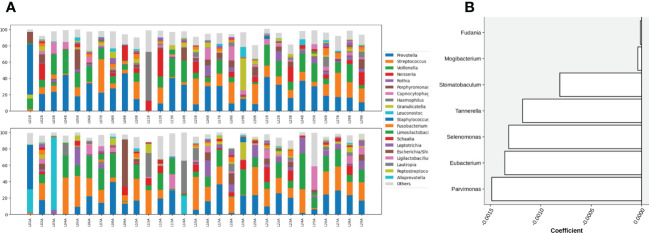
**(A)** Compositions of the top 20 abundant genera in oral samples of group L before (top) and after (bottom) gargling, and **(B)** differentially abundant genera. Note that the scale of coefficients is different from that in **Figure 3B**.

Substantial individual differences were again observed for patients in group L (**Figure 5A**). Paired analysis revealed that Firmicutes increased while Bacteroidetes, Spirochaetes, and Candidatus Saccharibacteria decreased after gargling (**Table S3B**). Seven differentially abundant genera were observed and all of them showed a decreased fraction after gargling (**Figure 5B**). Among the seven genera, only *Selenomonas* was also identified in group C. This indicates that bacterial species targeted by LIS were largely different from those by CHX. For the genera suppressed by LIS, the magnitudes of fractional changes were smaller compared to CHX (note the different scales of coefficients in **Figures 3B**, **5B**; the coefficients of *Selenomonas* were similar in the two). None of the seven differentially abundant genera were among the top 20 abundant ones. Therefore, the effect of LIS on the differentially abundant microbes was smaller compared to CHX. At the species level, only two species (*Corynebacterium matruchotii* and *Leptotrichia wadei*) showed different abundance after gargling (**Figure S3**). Like CHX, the genus level diversities decreased significantly after gargling LIS (**Figure 4B**).

### 3.5 Oral microbiota of the control group

**Figure 6:**
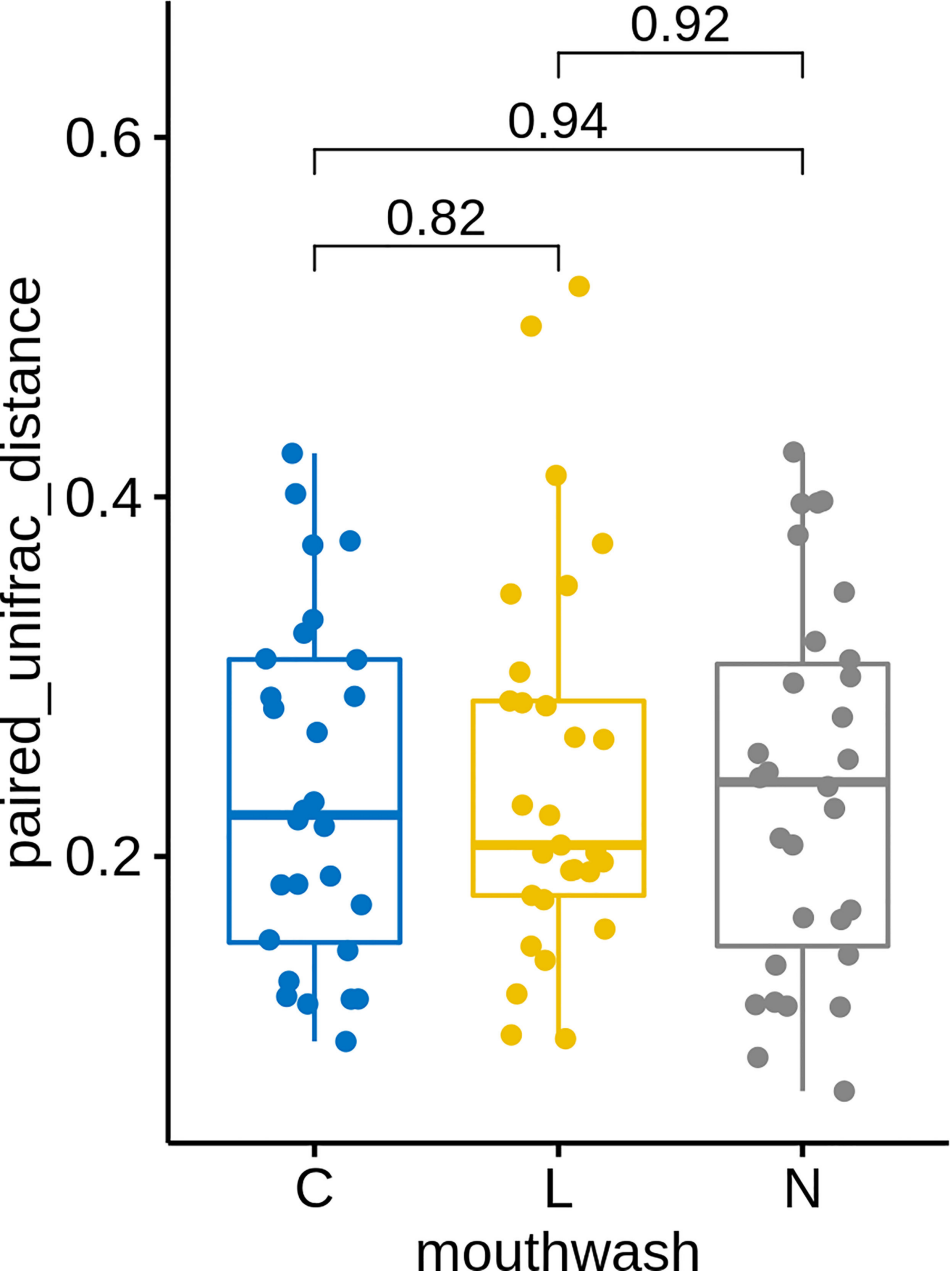
UniFrac distances between microbiota before and after gargling of the same individuals in three mouthwash groups. Differences between groups are tested by Wilcoxon rank test.

Similar to groups C and L, a large change in microbial composition was observed for several individuals in this control group (**Figure S4A**). In fact, the magnitudes of change in microbiota after gargling were not different for the three groups (**Figure 6**). This suggests that oral microbiota could be altered largely because of the act of gargling. Paired analysis revealed only one differentially abundant genus (**Figure S4B**), and the magnitude of change was relatively small. Taken together, only CHX and LIS resulted in consistent fractional changes of some bacterial genera. Gargling normal saline decreased the genus level diversities, but not significantly (**Figure 4C**).

### 3.6 Functional difference after gargling

Functional pathways in each microbial sample were predicted and the abundances of each pathway before and after gargling were compared for each individual. As the abundance change is bi-directional, we first selected differentially abundant (DA) pathways that showed a preferred direction of change (called consistent DA pathway) in all individuals of each mouthwash group. Of the 153 functional pathways, 26 and 6 were consistent DA in the groups C and L, respectively (**Table S4**). This is consistent with the observed smaller magnitudes of changes in microbial relative abundances after gargling LIS than CHX. There were no common consistent DA pathways in the two groups, suggesting different functional impacts of the CHX and LIS. No consistent DA pathway was found in the control group, which echoes the random alteration in microbial abundance after gargling normal saline. Several functional pathways (e.g., flagellar assembly, and lipopolysaccharide biosynthesis) were decreased after gargling CHX. This suggests a preference of CHX on targeting certain types of bacteria. Preference of LIS on target bacteria is also hinted in the pathway analysis. After gargling LIS, the relative abundance of “vitamin B6 metabolism” decreased and there was an increase of relative abundance of “glycerolipid metabolism” after gargling LIS (**Table S4**).

### 3.7 Microbial association with clinical features

Oral microbiota might be affected by physiological or pathological status of individuals, e.g., with drinking habit or diagnosis with cancer. Therefore, we searched for microbes associated with various clinical features. As the clinical features were expected to affect only the baseline levels of microbes, only samples before gargling were analyzed. Among the clinical features, a significant association was found for ten genera and eight of them were associated with alcohol consumption (**Figure S5**). One genus was associated with age and one was with smoking. None of the ten genera were differentially abundant for the three mouthwash groups. Thus, bacterial genera targeted by the mouthwashes differed from those associated with the clinical features. This supports our assumption that the clinical features only affect the baseline levels of microbes, while gargling was expected to pose a short-term impact.

## 4 Discussion

In this randomized clinical study on hospitalized patients, we explored the short-term effects of CHX mouthwash and LIS on salivary microbiome using a 16S approach. The short-term design was motivated by the usual length of hospital stay (Moloney et al., 2005), which is about one week, and is similar to several related studies (Tribble et al., 2019; Bescos et al., 2020). Compared to the *in vitro* studies, our 16S approach allows a more comprehensive and less biased investigation of oral microbes regarding the effects of mouthwash. To our knowledge, this is the first metagenomic study about the effect of LIS on salivary microbes. Including CHX mouthwash ensures a fair comparison of the two mouthwashes under the same experimental procedure. Including normal saline allows us to discern the effect of mouthwash from the act of gargling, thus strengthens validity of the identified bacteria targeted by the mouthwashes. In addition, paired analysis reduces noises from individual differences as many microbial taxa were not identified using unpaired analysis (data not shown).

In response to CHX mouthwash, our differentially abundant bacterial genera were consistent with the results of Bescos et al. (Bescos et al., 2020). Specifically, four of our eight decreased genera (*Prevotella*, *Fusobacterium*, *Campylobacter*, and *Corynebacterium*) were also decreased in the previous study, and *Prevotella* showed the largest decrease in both studies. Although not yet significant, *Rothia* had a high positive score in the previous work. The consistency is reasonable because we and Bescos et al. studied salivary microbiome using a 16S approach. Some of our differentially abundant genera had also been reported in previous *in vitro* studies. For example, *Prevotella* and *Selenomonas* were the two most suppressed bacteria by CHX in the study of McBain et al. (McBain et al., 2003), and the levels of both genera decreased in our study. Note that as the 16S approach only reveals relative abundance, it is possible that *Rothia* was also suppressed by the CHX mouthwash, but to a lesser degree compared to other genera, which ended up with an increased relative abundance.

Seven bacterial genera were found to be targeted by LIS, and only *Selenomonas* was also suppressed by the CHX mouthwash. This indicates that the bacterial species targeted by CHX and LIS were largely different. Of our seven genera targeted by LIS, *Parvimonas*, *Eubacterium*, and *Tannerella* were also mentioned in a previous study where bacterial levels were quantified using PCR (Hwang et al., 2021). The most suppressed *Parvimonas* has also been reported to be suppressed by LIS in a previous *in vitro* study (Filipovic et al., 2020).

While both the CHX mouthwash and LIS targeted certain bacterial genera, the magnitudes of changes in relative abundance were smaller using LIS in general. This suggests a milder effect of LIS against the target microbes. However, we note that as the 16S approach only reveals relative abundance, the effects in absolute abundance cannot be inferred. CHX has been shown to suppress oral bacteria more effectively than LIS (Haffajee et al., 2008; Thomas et al., 2015), but the higher efficiency is still controversial (Quintas et al., 2015). LIS are generally considered less harmful than CHX (Tsourounakis et al., 2013), however, the statement varies between different settings (Park et al., 2014). Although the physiological impact of mouthwashes still needs further investigation, their functional role can be inferred *via* the identified target bacteria.

The dysbiosis of the upper respiratory tract microbiome has been associated with pneumonia (de Steenhuijsen Piters et al., 2016). In oropharyngeal microbiota, the genera *Rothia* and *Prevotella* were found more and less abundant in pneumonia patients respectively compared to healthy individuals. The two genera showed the largest change in relative abundance, with *Rothia* increased while *Prevotella* decreased, after gargling CHX. Therefore, gargling CHX likely moved the oral microbiota toward the profiles in pneumonia patients. Several *Prevotella* species (*P. melaninogenica, buccae, tannerae, and nanceiensis*) in the airway have been associated with the clearance of *Streptococcus pneumoniae* (Horn et al., 2022), a bacterial pathogen causing community-acquired pneumonia. *P. melaninogenica* was the most abundant *Prevotella* species in our data. However, its relative abundance did not change significantly after gargling CHX, neither did the three other *Prevotella* species. In our hospitalized patients, *P. nigrescens* was decreased after gargling CHX, but it was not associated with the clearance of *S. pneunomiae*. Therefore, the impact of CHX on pneumonia *via* altering oral microbes still needs further investigation.

Oral nitrate‐reducing bacteria are beneficial to vascular health and linked to blood pressure regulation and insulin resistance (Goh et al., 2019; Pignatelli et al., 2020). In our hospitalized patients, *Prevotella* spp. were reduced the most after gargling the CHX mouthwash while not suppressed after gargling LIS and normal saline. *Prevotella* is the most abundant genus of oral nitrate-reducing bacteria in healthy adults (Burleigh et al., 2018). Consistently, CHX mouthwash eliminated nitrate-nitrite conversion markedly, which resulted in lower plasma and salivary concentration of nitrite, while no difference was found after gargling LIS (Woessner et al., 2016). Other oral nitrate-reducing bacteria include *Neisseria*, *Rothia*, *Veillonella*, *Antinomyces*, *Corynebactrium*, and *Haemophilus* (Rosier et al., 2020), among which *Corynebacterium* were decreased and *Rothia* were increased in our hospitalized patients after gargling the CHX mouthwash. Many of the mentioned nitrate-reducing bacteria were not reduced after gargling LIS. A common genus reduced by CHX and LIS in our data was *Selenomonas*, which capability to reduce nitrate has been reported (Asanuma et al., 2015). The degrees of suppression using the two mouthwashes were similar. Taken together, LIS could also reduce some nitrate-reducing bacteria. However, CHX targeted more nitrate-reducing bacteria and to a greater degree.

In a previous study (de Steenhuijsen Piters et al., 2016), the domination of *Rothia* was strongly associated with pneumonia. And iron-chelating enterobactin might explain *Rothia mucilaginosa*’s successful oral colonization in both healthy and diseased individuals (Uranga et al., 2020). However, a recent literature showed that *Rothia mucilaginosa* abundance inversely correlated with sputum pro-inflammatory markers in chronic lung disease, which implies a beneficial role in the respiratory tract (Rigauts et al., 2022). Therefore, the role of *Rothia mulcilaginosa* in pneumonia is still controversial.

Our pathway analysis reveals a decreased relative abundance in several bacterial activities after gargling CHX, e.g., bacterial chemotaxis, flagellar assembly, and lipopolysaccharide (LPS) biosynthesis. This suggests preference of CHX on targeting certain types of bacteria. For example, CHX may prefer to target gram-negative bacteria because LPS is a major component of their outer membrane. Indeed, CHX binds to LPS and suppresses the LPS-induced inflammation after the bacteria are destroyed (Zorko and Jerala, 2008). Preference of LIS on target bacteria is also hinted in the pathway analysis. After gargling LIS, the relative abundance of “vitamin B6 metabolism” decreased. Many bacteria in the Bacteroidetes phylum possess a vitamin B6 biosynthesis pathway, while most Firmicutes lack such the pathway (Yoshii et al., 2019). After gargling LIS, Bacteroidetes decreased while Firmicutes increased, which could explain the decreased “vitamin B6 metabolism”.

We also observed an increase of relative abundance of “glycerolipid metabolism” after gargling LIS. This may also suggest a preference of LIS on target bacteria because bacterial membranes are diverse in the contents of various lipids (Sohlenkamp and Geiger, 2016). However, we give a caveat to the functional interpretation. Glycerolipid is also a major component of plant membrane (Reszczynska and Hanaka, 2020) and could be in the essential oil. Therefore, bacteria might engage in processing the glycerolipid in the LIS. Similarly, the increase of “sulfur metabolism” after gargling LIS seems reasonable because volatile sulfur compounds produced by bacteria are the source of oral malodor (Foo et al., 2021) and LIS may target those bacteria. However, CHX has been shown to reduce volatile sulfur compounds more effectively than essential oil mouthwash (Malhotra and Yeltiwar, 2011), but the pathway was not consistently different after gargling CHX.

We examined the correlation between the baseline (i.e., before gargling) oral microbes and the clinical features, including alcohol drinking and smoking habits. The correlation analysis was performed using multiple regression, that is, all variables were examined simultaneously. The result suggests a correlation between some genera and alcohol drinking, smoking, and age. No correlation was observed for all other variables. Therefore, we found no bacterial genera associated with BMI, diabetes, cancer, cirrhosis, infections, and end-stage renal disease. In our analysis of the correlation between baseline (i.e., before gargling) bacterial genera and alcohol drinking, we found that *Desulfobulbus* was more abundant in alcohol users than non-alcoholic individuals. A similar finding was reported (Barb et al., 2022). As for the smoking-associated *Bulleidia*, the genus was also reported to be more abundant in smoking than non-smoking individuals (Jia et al., 2021). The *Parascardovia* genus was found associated with age in our study. Parascardovia has been shown to be predominant in diabetic salivary microbiome in another study (Liu et al., 2021). But the association was not found in the elderly in the published literature.

There are limitations of this study. First, an observed alteration in relative abundance did not guarantee a change in absolute abundance in the same direction. Therefore, one needs to be cautious about linking the altered microbiota to potential functional impacts, e.g., the susceptibility to pneumonia and nitrate reduction, which were not measured in this work. Second, we did not intervene the patients’ oral cleaning such as toothbrushing and the toothpaste use, which might impact the oral microbiome (Chhaliyil et al., 2020; Shang et al., 2020). However, we expect that toothpaste use and brushing method posed a long-term impact on the baseline (i.e., before gargling) oral microbiota in the recruited individuals. In this study, we examined the short-term effects of mouthwashes on oral microbiota. By checking microbial differences before and after gargling, the baseline microbiota only served as a reference point. We thus do not expect that the long-term habit of toothpaste use and brushing method posed a noticeable impact on the short-term alteration in oral microbiota. In a previous study, short term use (~2 weeks) of toothpastes with different active components did not lead to different relative abundance of the dominating genera in oral microbiome (Shang et al., 2020). Although we did not control the toothpaste use and brushing method, the random assignment of subjects to groups should alleviate the impact if there is any. Besides, the consistent findings with the literature suggests a good randomization. Third, it may not be appropriate to extrapolate our findings for long-term alteration of oral microbiota. For example, no differentially abundant oropharyngeal bacteria were observed after using LIS for 12 weeks. It is possible that certain bacteria develop resistance to mouthwash in the long run (Cieplik et al., 2019). Therefore, long-term effects of mouthwash on oral microbiome still need further investigation. Fourth, neither a dentist nor a stomatologist performed a clinical examination and registered the oral hygiene indices. And the sole “inspection” of the oral cavity without the proper light, instruments, and operator can underestimate any differences in the baseline oral conditions. This might lead to variations in baseline oral hygiene among the recruited individuals. However, the potential difference between groups of individuals could be addressed by randomization.

In conclusion, this is the first metagenomic study about the effects of LIS on oral microbes. Both the CHX mouthwash and LIS altered oral microbiota in our hospitalized patients. Bacterial genera targeted by the CHX mouthwash and LIS were largely different, and the magnitude of suppression by LIS was also smaller. Moreover, fewer bacterial genera targeted by LIS were reported to be nitrate-reducing compared to CHX mouthwash.

## Data availability statement

The datasets presented in this study can be found in online repositories. The names of the repository/repositories and accession number(s) can be found below: https://www.ncbi.nlm.nih.gov/, PRJNA834608.

## Ethics statement

The studies involving human participants were reviewed and approved by Institutional review board of the National Cheng Kung University Hospital (no: A-ER-108-397). The patients/participants provided their written informed consent to participate in this study.

## Author contributions

Y-CC, TL, and J-LW: conceptualization. TL, S-LJ, and J-JC: methodology. K-NY and W-CK: validation C-HL and TL: formal analysis. Y-CC, P-FT and J-LW: investigation. Y-CC and J-LW: writing—original draft preparation. TL, and W-CK: writing—review and editing. J-YW and K-NY: supervision. All authors have read and agreed to the published version of the manuscript
