# Supplementary material for: Short-term effects of Chlorhexidine mouthwash and Listerine on oral microbiome in hospitalized patients
Source: Front Cell Infect Microbiol. 2023 Feb 2;13:1056534. doi: 10.3389/fcimb.2023.1056534 (PMC9932516; doi:10.3389/fcimb.2023.1056534)
Supplement: Supplementary file 1 [file DataSheet_1.pdf]

SUPPLEMENTARY MATERIALS

Supplementary Tables

PID	sex	age	smoking	alcohol	BMI	diabetes	cancer	cirrhosis	ESRD	infection
C01	F	67	No	No	28.00	Yes	No	No	No	No
C02	F	66	No	No	24.90	No	Yes	No	No	No
C03	M	24	No	No	19.44	No	No	No	No	No
C04	M	63	No	No	30.57	No	No	No	No	No
C05	M	55	No	No	29.46	No	No	No	No	No
C06	F	42	No	No	37.73	No	No	No	No	No
C07	F	76	No	No	23.60	No	No	No	No	Yes
C08	M	78	No	No	17.99	Yes	No	No	Yes	No
C09	F	67	No	No	29.20	Yes	No	No	No	Yes
C10	M	78	No	No	20.95	No	No	No	No	No
C11	M	52	Yes	Yes	30.34	No	No	No	No	Yes
C12	M	76	No	No	21.18	No	Yes	No	No	Yes
C13	F	58	No	No	20.77	Yes	No	No	No	Yes
C14	M	61	No	No	26.95	No	No	No	No	Yes
C15	F	79	No	No	35.26	Yes	No	No	No	Yes
C16	F	72	No	No	16.67	No	Yes	No	Yes	Yes
C17	M	70	No	No	29.30	No	No	No	No	Yes
C18	F	87	No	No	23.05	Yes	No	Yes	No	Yes
C19	M	52	Yes	No	22.69	No	Yes	No	No	Yes
C20	M	62	No	No	22.23	Yes	Yes	Yes	No	No
C21	M	70	No	No	29.24	No	No	No	No	Yes
C22	M	53	No	No	25.65	Yes	No	No	No	No
C23	M	78	Yes	No	25.86	No	No	No	No	No
C24	M	59	No	No	28.65	No	Yes	Yes	No	No
C25	M	65	No	Yes	30.76	Yes	No	No	No	No
C26	F	83	No	No	24.64	No	No	No	No	Yes
C27	M	62	Yes	No	17.27	Yes	No	No	No	No
C28	M	66	No	Yes	29.32	Yes	Yes	No	No	Yes
L01	M	84	No	No	21.95	No	No	Yes	No	Yes
L02	F	64	No	No	24.55	No	No	No	No	Yes
L03	F	45	Yes	No	21.38	No	No	No	No	No
L04	F	64	No	No	23.29	Yes	No	No	No	No
L05	M	59	Yes	No	28.62	No	No	No	No	Yes
L06	F	72	No	No	23.15	No	No	No	No	No
L07	M	76	No	No	21.77	Yes	No	No	No	No
L08	M	61	No	No	26.91	No	No	Yes	No	No
L09	M	83	No	No	23.57	No	No	No	No	Yes
L10	F	60	Yes	No	22.13	No	Yes	No	No	Yes
L11	M	62	No	No	22.46	No	No	No	No	No
L12	M	60	Yes	No	27.00	Yes	Yes	No	No	No
L13	M	68	Yes	No	26.20	Yes	No	No	No	Yes
L14	F	55	No	No	21.76	Yes	No	No	No	Yes
L15	M	54	Yes	No	28.33	Yes	No	No	No	No
L16	M	54	No	Yes	23.69	Yes	No	No	No	No
L17	M	62	No	No	22.98	No	No	No	No	Yes
L18	F	69	No	No	29.92	Yes	No	No	No	No
L19	M	59	Yes	No	25.21	No	No	Yes	No	Yes
L20	M	66	No	No	16.44	No	Yes	Yes	No	Yes
L21	M	61	No	No	23.01	No	No	No	No	No
L22	M	63	No	No	23.91	Yes	No	No	No	No
L23	F	78	No	No	20.53	No	No	No	No	No
L24	F	69	No	No	20.42	No	No	No	No	Yes
L25	F	58	No	No	25.83	Yes	Yes	Yes	No	No
L26	M	63	No	No	26.30	No	No	No	No	Yes
L27	F	61	No	No	25.92	No	No	No	No	No
L28	M	70	No	No	25.47	Yes	No	No	No	No

L29	M	71	No	No	33.00	No	Yes	Yes	No	Yes
N01	M	67	No	No	20.61	Yes	No	No	No	No
N02	F	58	No	No	25.36	Yes	No	No	No	No
N03	F	60	No	No	33.01	No	Yes	No	No	No
N04	F	79	No	No	20.94	No	No	No	No	No
N05	F	78	No	No	26.83	Yes	Yes	No	No	No
N06	F	52	Yes	Yes	22.85	Yes	No	No	No	Yes
N07	M	58	No	No	36.11	Yes	No	Yes	No	No
N08	F	55	No	No	19.82	Yes	No	No	Yes	No
N09	M	66	Yes	Yes	29.58	No	No	No	No	No
N10	F	61	No	No	23.50	No	Yes	No	No	Yes
N11	M	45	Yes	No	15.06	No	No	No	No	No
N12	M	55	No	No	29.52	No	No	No	No	Yes
N13	M	76	No	No	23.52	Yes	No	No	No	Yes
N14	F	82	No	No	23.49	Yes	Yes	No	No	Yes
N15	M	52	No	No	20.22	No	Yes	No	No	Yes
N16	F	78	No	No	22.51	Yes	No	Yes	No	Yes
N17	F	74	No	No	26.53	No	No	No	No	Yes
N18	M	56	No	No	25.19	Yes	No	No	No	Yes
N19	M	89	No	No	24.29	No	No	No	No	No
N20	F	80	No	No	27.53	No	No	No	No	Yes
N21	M	66	No	No	27.49	No	No	No	No	No
N22	F	54	No	No	29.63	No	No	No	No	No
N23	M	73	Yes	No	23.23	Yes	Yes	No	No	No
N24	F	73	No	No	26.33	No	No	No	No	Yes
N25	M	57	No	No	22.03	No	No	No	No	Yes
N26	F	60	No	No	30.13	Yes	No	No	No	Yes
N27	M	51	No	No	29.72	No	No	No	No	Yes
N28	F	50	No	No	32.32	Yes	No	No	No	Yes
N29	F	65	No	No	21.77	No	No	No	Yes	No
N30	M	65	No	No	23.70	Yes	No	No	No	Yes

Table S1. Clinical features of recruited individuals in the three mouthwash groups (PID starting with C, L, and N, respectively).

Sample	Raw PE reads	Merged reads	Long merged reads	Aligned and filtered reads
C01A	57508	55133	55103	49901
C01B	62804	60042	60006	53312
C02A	66544	63608	63498	58185
C02B	90964	86544	86392	78163
C03A	90919	87228	87096	79361
C03B	78865	75236	74988	67887
C04A	56398	52628	51464	47290
C04B	60942	57551	57463	51844
C05A	57737	55422	55231	50918
C05B	56805	54561	54515	50071
C06A	53705	51003	50517	47060
C06B	56266	53525	53094	49000
C07A	43574	41282	40642	37535
C07B	55815	53241	53164	48524
C08A	54843	52357	52338	47620
C08B	54489	52049	52034	47233
C09A	80571	78094	77762	71503
C09B	113958	109745	109254	99844
C10A	91061	87888	87765	80333
C10B	99985	96381	96271	87931
C11A	62927	60625	60580	56650
C11B	85778	81751	81537	74980
C12A	118974	114028	113358	105586
C12B	78974	74455	73549	68457
C13A	87905	84274	84155	78256

C13B	73615	70709	70622	65946
C14A	56730	54250	53385	49809
C14B	57980	55477	55419	51361
C15A	72873	70318	70223	64285
C15B	86876	83931	83859	77728
C16A	86477	82677	82616	75287
C16B	102694	98155	98097	88678
C17A	114793	108452	108181	97728
C17B	87247	82586	82555	75853
C18A	105233	100550	99320	87821
C18B	136679	131167	131091	119181
C19A	72528	69034	68350	65208
C19B	90856	87625	86999	75119
C20A	108742	104359	104331	93752
C20B	122084	116209	116185	101349
C21A	71643	68666	68637	62933
C21B	107939	102717	102631	93568
C22A	75119	72791	72508	68275
C22B	106412	102506	102326	93801
C23A	76721	73396	73161	68135
C23B	80924	77511	77001	71309
C24A	78461	74490	74242	66910
C24B	99443	95594	95341	85340
C25A	91129	86906	86819	78806
C25B	84383	80818	80691	73658
C26A	84103	80403	79996	74769
C26B	70473	67512	67217	64461
C27A	85954	82072	81676	74816
C27B	84831	81240	81177	70704
C28A	69835	66120	63963	59964
C28B	71454	68338	67461	62887
L01A	47469	45672	45527	41643
L01B	73794	70356	70201	65632
L02A	71315	67862	67834	59969
L02B	69776	66463	66450	58747
L03A	62981	60153	59900	56980
L03B	64013	60947	60928	53220
L04A	63979	60904	60886	55580
L04B	56334	53742	53698	49111
L05A	56508	53052	52988	48837
L05B	58913	56265	56229	51787
L06A	61467	58819	58673	53756
L06B	60192	56849	56503	51441
L07A	59375	56356	56285	50673
L07B	42453	40563	40525	37049
L08A	54356	51921	51874	47588
L08B	63922	60550	60475	54522
L09A	81258	77084	74755	68858
L09B	80499	77018	76364	71620
L10A	100877	97066	97006	88879
L10B	101076	96960	96905	88668
L11A	89847	86373	86320	79068
L11B	65773	63062	62982	57102
L12A	93410	89117	88562	80310
L12B	87549	83886	83772	76287
L13A	71346	68690	68164	62589
L13B	79692	76425	76251	69660
L14A	62247	59115	58519	53139
L14B	93117	89191	89099	80869
L15A	66980	64137	64108	59000
L15B	55595	53178	53157	49126
L16A	85846	81303	81236	74515
L16B	80996	77592	77515	70812
L17A	114359	108579	108549	99995

L17B	98117	93081	93006	85909
L18A	149160	141358	141269	130214
L18B	92610	87905	87834	81262
L19A	89181	85179	84997	75320
L19B	61759	58894	58147	54665
L20A	147321	139150	139134	126643
L20B	149333	143141	143093	127404
L21A	94609	91637	91539	83524
L21B	73457	71050	70814	65738
L22A	81683	78264	78201	70642
L22B	101101	97095	96907	83653
L23A	109204	105156	104954	92670
L23B	93133	89965	89889	81886
L24A	72054	67480	66941	64042
L24B	77252	73728	73198	66941
L25A	74079	71635	71432	64133
L25B	79560	76540	76487	68751
L26A	77129	73352	73281	66665
L26B	86542	82528	82193	74914
L27A	75465	71706	71563	65965
L27B	83302	78547	78068	73234
L28A	79336	76452	76370	68604
L28B	108405	103100	103009	93730
L29A	77888	74151	74049	66238
L29B	79983	76963	76835	68843
N01A	78125	74841	74816	67519
N01B	68512	65723	65554	60342
N02A	76510	71903	70492	64073
N02B	71096	67716	67595	61392
N03A	80148	76953	76841	70260
N03B	89794	85547	85425	76647
N04A	69991	66980	66951	58428
N04B	65173	62276	62255	53456
N05A	58633	56306	56299	50713
N05B	52975	50416	50410	44609
N06A	60618	57857	56979	51857
N06B	65418	61888	61527	56422
N07A	73559	69895	69655	64149
N07B	61157	58221	58042	53488
N08A	58013	55279	55234	50101
N08B	54807	52076	52054	47172
N09A	76059	72206	72096	65688
N09B	63122	60167	60137	54299
N10A	97331	93928	93870	85979
N10B	102526	98928	98790	90030
N11A	121933	117718	117662	107786
N11B	70150	67317	66962	63073
N12A	68521	65457	64935	60954
N12B	71302	67478	65911	61897
N13A	105679	101618	101505	93847
N13B	106397	101685	101496	93499
N14A	61937	57790	56993	52268
N14B	93655	88338	87022	80280
N15A	79205	75332	74833	67039
N15B	102830	98216	97335	89125
N16A	105717	100858	100300	90225
N16B	90702	86220	85529	78791
N17A	68062	65032	65004	58902
N17B	68724	65396	65352	59106
N18A	69719	66629	66557	61736
N18B	115950	110246	110067	100297
N19A	71787	68726	68703	63251
N19B	91065	86640	86335	79541
N20A	100629	96670	96572	87108

N20B	129179	123480	123423	108612
N21A	110504	105851	105826	97708
N21B	103887	98997	98950	89121
N22A	117812	113187	113136	101835
N22B	104591	100400	100351	91697
N23A	83080	79889	79788	73156
N23B	84691	81716	81658	75225
N24A	79622	76023	75816	72444
N24B	87145	83289	82642	75061
N25A	74419	71298	71139	66781
N25B	87728	83271	80919	72019
N26A	95932	91964	91883	82953
N26B	97944	94303	94225	83925
N27A	79686	76494	76421	70580
N27B	66144	62969	62248	58577
N28A	67479	64444	64016	58016
N28B	81815	78502	78368	70764
N29A	83360	80271	80249	74479
N29B	83392	80452	80353	73545
N30A	79022	75779	75642	67208
N30B	74528	71585	71460	63991
Total	14181010	13553173	13505914	12318553

Table S2. Statistics of sequencing reads.

phylum	coefficient	q-value
(a)		
Fusobacteria	-0.0112	0.0076
Campilobacterota	-0.0021	0.0325
Candidatus.Saccharibacteria	-0.0011	0.0325
Bacteroidetes	-0.0319	0.0612
(b)		
Firmicutes	0.0481	0.0211
Bacteroidetes	-0.0528	0.0211
Spirochaetes	-0.0029	0.0829
Candidatus Saccharibacteria	-0.0014	0.0829

Table S3. Differentially abundant phyla after gargling (a) CHX and (b) LIS by MaAsLin2.

pathway	lower	higher	p-value	q-value
(a)				
Epithelial cell signaling in Helicobacter pylori infection	15	0	0.00006	0.00934
Aminobenzoate degradation	3	19	0.00086	0.03937
African trypanosomiasis	4	21	0.00091	0.03937
D-Glutamine and D-glutamate metabolism	16	2	0.00131	0.03937
RNA polymerase	18	3	0.00149	0.03937
Glutathione metabolism	4	20	0.00154	0.03937
Spliceosome	15	2	0.00235	0.04972
Steroid hormone biosynthesis	19	4	0.00260	0.04972
NOD-like receptor signaling pathway	18	4	0.00434	0.06155
Amoebiasis	16	3	0.00443	0.06155
Pantothenate and CoA biosynthesis	16	3	0.00443	0.06155
One carbon pool by folate	19	5	0.00661	0.08429
Fluorobenzoate degradation	2	13	0.00739	0.08692
Bacterial chemotaxis	20	6	0.00936	0.08946
Flagellar assembly	20	6	0.00936	0.08946
Lipopolysaccharide biosynthesis	20	6	0.00936	0.08946

Polycyclic aromatic hydrocarbon degradation	4	16	0.01182	0.10636
Plant-pathogen interaction	12	2	0.01294	0.10999
Riboflavin metabolism	19	6	0.01463	0.11243
Biosynthesis of unsaturated fatty acids	5	17	0.01690	0.11243
Pyruvate metabolism	5	17	0.01690	0.11243
Terpenoid backbone biosynthesis	17	5	0.01690	0.11243
Tryptophan metabolism	5	17	0.01690	0.11243
Photosynthesis - antenna proteins	3	13	0.02127	0.13148
Endocytosis	9	1	0.02148	0.13148
Two-component system	5	16	0.02660	0.15655
(b)				
Glycerolipid metabolism	4	18	0.00434	0.18593
Vitamin B6 metabolism	18	4	0.00434	0.18593
Sulfur metabolism	6	21	0.00592	0.18593
Photosynthesis	5	19	0.00661	0.18593
Selenocompound metabolism	5	19	0.00661	0.18593
Carbon fixation in photosynthetic organisms	17	4	0.00720	0.18593

Table S4. Consistent differentially abundant pathways in mouthwash groups (a) C and (b) L. Columns 2 and 3 indicate numbers of individuals showing a lower and higher pathway abundance after gargling the mouthwash. Columns 4 and 5 show the binomial test p-values and the corrected q-values.

Supplementary Figures

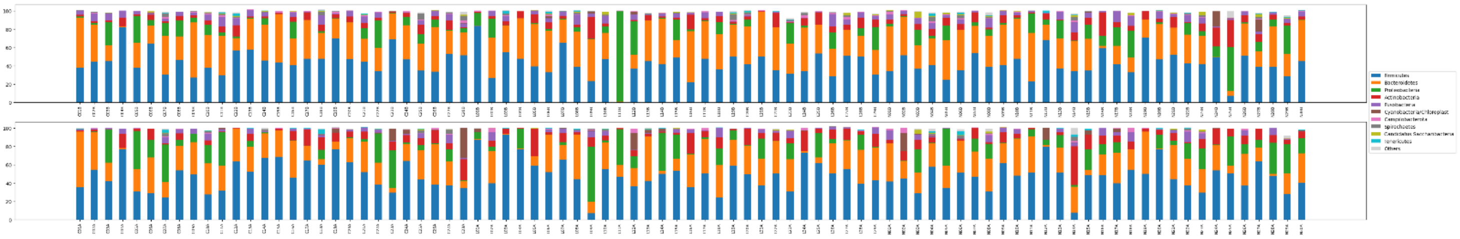

Figure S1. Compositions of top ten abundant phyla in all samples before (top) and after (bottom) gargling. Samples of three mouthwash groups C, L, and N are placed on the left, middle, and right.

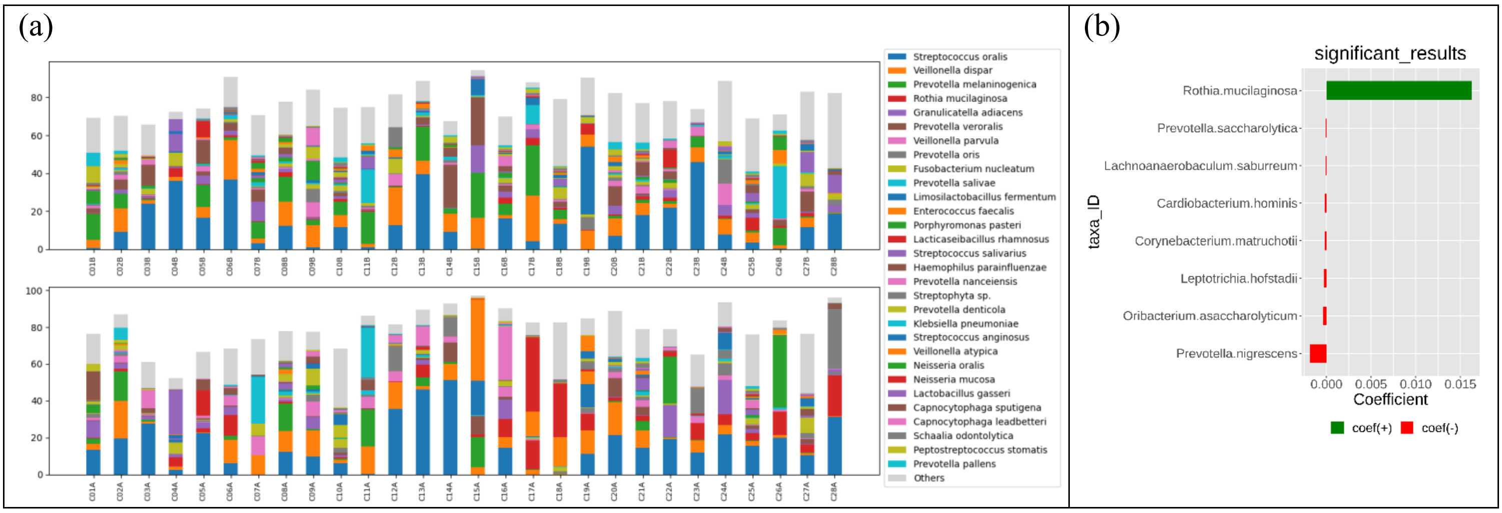

Figure S2. (a) Compositions of top 30 abundant species in oral samples of group C before (top) and after (bottom) gargling, and (b) differentially abundant species.

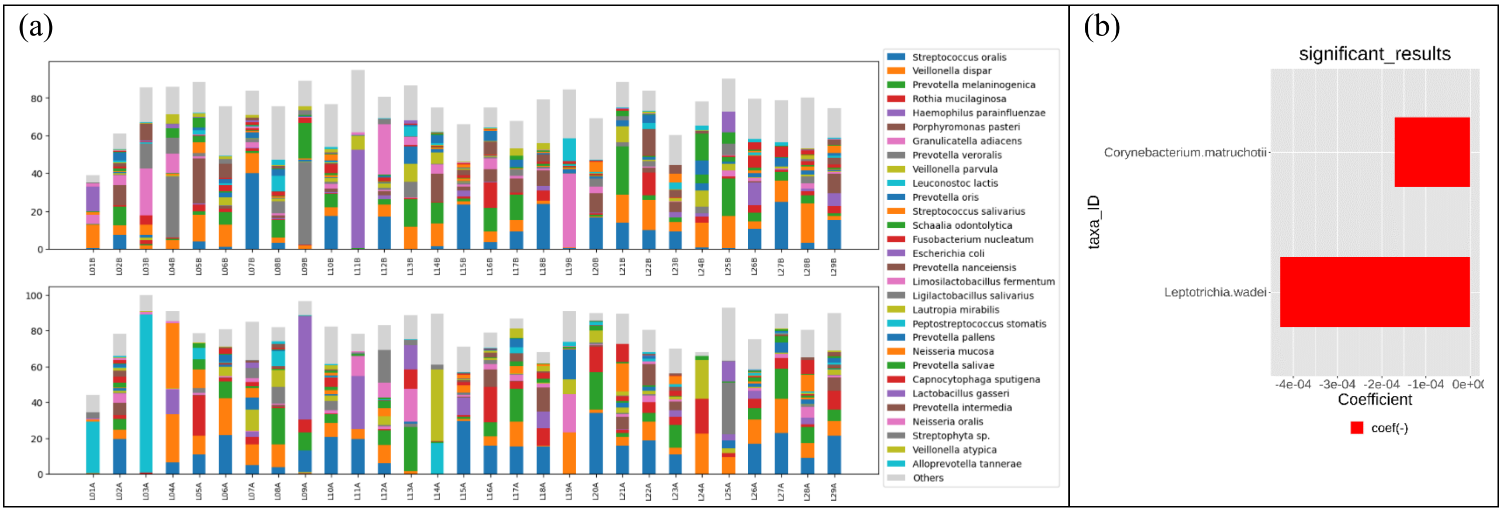

Figure S3. (a) Compositions of top 30 abundant species in oral samples of group L before (top) and after (bottom) gargling, and (b) differentially abundant species.

(a)	(b)

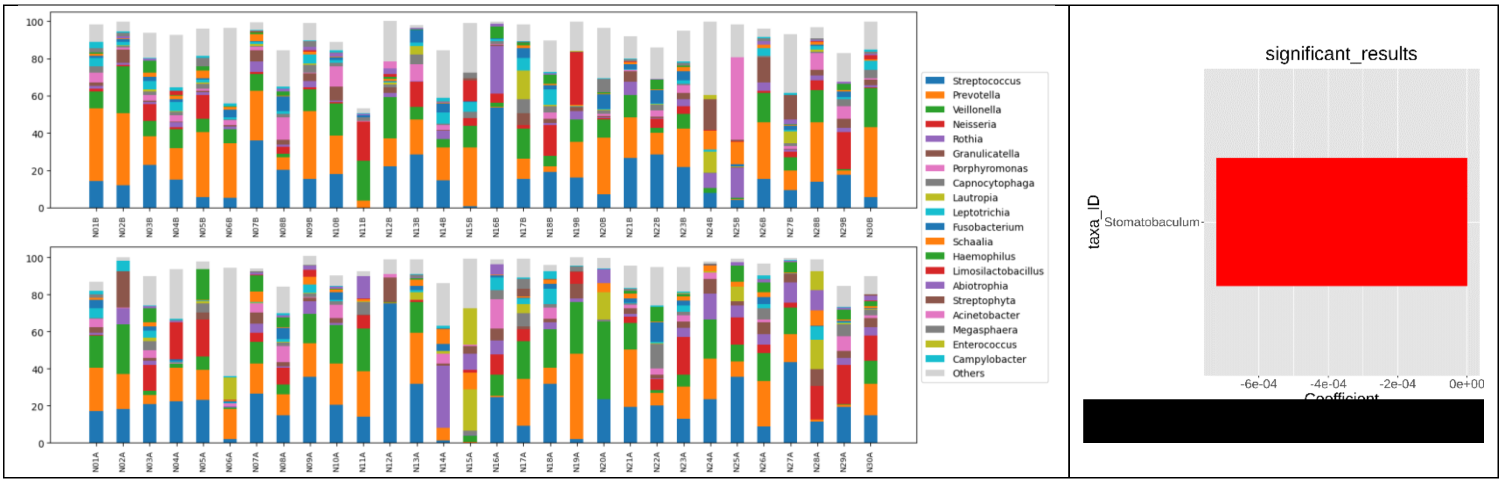

Figure S4. (a) Compositions of top 20 abundant genera in oral samples of group N before (top) and after (bottom) gargling, and (b) differentially abundant genus.

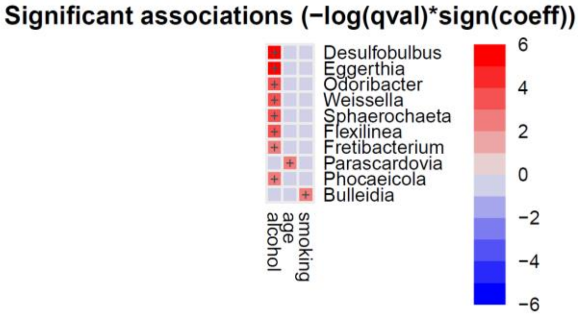

Figure S5. Microbial genera associated with clinical features.
